# A combined analysis of genetically correlated traits identifies 187 loci and a role for neurogenesis and myelination in intelligence

**DOI:** 10.1038/s41380-017-0001-5

**Published:** 2018-01-11

**Authors:** W. D. Hill, R. E. Marioni, O. Maghzian, S. J. Ritchie, S. P. Hagenaars, A. M. McIntosh, C. R. Gale, G. Davies, I. J. Deary

**Affiliations:** 1 Centre for Cognitive Ageing and Cognitive Epidemiology, University of Edinburgh, Edinburgh, UK; 2 Department of Psychology, University of Edinburgh, Edinburgh, UK; 3 Queensland Brain Institute, The University of Queensland, Brisbane, 4072 QLD Australia; 4 Department of Economics, Harvard University, Littauer Center, 1805 Cambridge Street Cambridge, Cambridge, MA 02138 USA; 5 MRC Social, Genetic and Developmental Psychiatry Centre, Institute of Psychiatry, King’s College London, Camberwell,, London SE5 8AF UK; 6 Division of Psychiatry, University of Edinburgh, Edinburgh, EH8 9YL UK; 7 MRC Lifecourse Epidemiology Unit, University of Southampton, Southampton, UK

**Keywords:** Psychology, Genetics

## Abstract

Intelligence, or general cognitive function, is phenotypically and genetically correlated with many traits, including a wide range of physical, and mental health variables. Education is strongly genetically correlated with intelligence (*r*_*g*_ = 0.70). We used these findings as foundations for our use of a novel approach—multi-trait analysis of genome-wide association studies (MTAG; Turley et al. 2017)—to combine two large genome-wide association studies (GWASs) of education and intelligence, increasing statistical power and resulting in the largest GWAS of intelligence yet reported. Our study had four goals: first, to facilitate the discovery of new genetic loci associated with intelligence; second, to add to our understanding of the biology of intelligence differences; third, to examine whether combining genetically correlated traits in this way produces results consistent with the primary phenotype of intelligence; and, finally, to test how well this new meta-analytic data sample on intelligence predicts phenotypic intelligence in an independent sample. By combining datasets using MTAG, our functional sample size increased from 199,242 participants to 248,482. We found 187 independent loci associated with intelligence, implicating 538 genes, using both SNP-based and gene-based GWAS. We found evidence that neurogenesis and myelination—as well as genes expressed in the synapse, and those involved in the regulation of the nervous system—may explain some of the biological differences in intelligence. The results of our combined analysis demonstrated the same pattern of genetic correlations as those from previous GWASs of intelligence, providing support for the meta-analysis of these genetically-related phenotypes.

Intelligence, also known as general cognitive function or simply *g*, describes the shared variance that exists between diverse measures of cognitive ability [1]. In a population with a range of cognitive ability, intelligence accounts for around 40% of the variation between individuals in scores on diverse cognitive tests [2]. Intelligence is predictive of health states, including mortality; [3, 4] a lower level of cognitive function in youth is associated with earlier death over the next several decades [5]. Intelligence is a heritable trait, with twin- and family-based estimates of heritability indicating that between 50–80% of differences in intelligence can be explained by genetic factors [6]. These genetic factors make a greater contribution to phenotypic differences as age increases from childhood to adulthood [7]. Heritability estimates derived from molecular genetic data using the GREML-SC [8, 9] method indicate that around 20–30% of variation can be explained by variants in linkage disequilibrium (LD) with genotyped single nucleotide polymorphisms (SNPs) [10]. Some of the association between intelligence and health is due to genetic variants that act across traits [11, 12]. More recent methods to measure heritability, such as GREML-KIN [13], and GREML-MS [14] using imputed SNPs, have found that some of the heritability of intelligence can be found in variants that are in poor LD with genotyped variants; by taking these into consideration, SNP heritability estimates of 0.54 (GREML-KIN) and 0.50 (GREML-MS) [15] have been found.

Relatively few genetic variants have reliably been associated with intelligence differences [16]. The sparsity of genome-wide significant SNPs discovered so far, combined with the substantial heritability estimate, suggests a phenotype with a highly polygenic architecture, where the total effect of all associated variants is substantial, but in which each individual variant exerts only a small influence. This is compelling evidence that the number of uncovered genome-wide significant loci associated with intelligence can be increased by raising the sample size—and thus the statistical power—of GWASs, as has been the case for other phenotypes such as height [17] and schizophrenia [18].

Two strategies have emerged in order to maximise power by increasing the sample size for loci discovery in intelligence research. The first involves the meta-analysis of many GWASs conducted on intelligence [19–21]. However, these studies are hampered by the fact that each individual sample tends to use different cognitive tests, and these individual sample sizes are often small; thus, even the resulting meta-analysis is underpowered to detect loci associated with intelligence with very small effect sizes [19–21]. This problem is ameliorated in studies like UK Biobank, which contain a large number of individuals who have supplied genetic data and taken the same cognitive test [22]. In the case of UK Biobank, a test of verbal and numerical reasoning shows a high genetic correlation with intelligence [23] as derived from psychometrically validated test batteries [16].

The second method is to use a “proxy” phenotype [24] that shows high phenotypic and genetic correlations with intelligence, and should therefore have a similar genetic architecture. Educational attainment has been successfully used as a proxy phenotype for intelligence [24], owing in part to the phenotypic and genetic correlation between the traits [7], and to the ease with which it can be measured relatively consistently, facilitating the larger sample sizes required for loci discovery [25]. Such methods have led to sample sizes of 293,723 for educational attainment, and the discovery of 74 loci attaining genome-wide significance [25]. The genetic correlation between the largest GWAS on intelligence and the largest GWAS on education was 0.70 [16].

In the present study, we combined these two approaches by using MTAG [26], a newly-developed technique that allows the meta-analysis of summary statistics from genetically-related traits. This enabled us effectively to increase the sample size (to add power) to GWASs of intelligence by adding in the genetic variance that is shared with proxy phenotypes. We used summary results from the largest available GWAS on intelligence (*n* = 78,308) [16]. We performed a meta-analysis using these data, and those from the latest release of the genetic data from UK Biobank to maximise power in our GWAS of intelligence. Finally, we added the Social Science Genetic Association Consortium (SSGAC) GWAS summary results for years of education [25] (*n* = 329,417, which include individuals from UK Biobank).

By combining our meta-analytic dataset on intelligence with the education dataset from the SSGAC, we increased the power to discover loci associated with intelligence. The estimated effective sample size increased from 199,242 to 248,482 participants. We then used bivariate linkage disequilibrium score regression [12] to test whether these meta-analytic results have the same genetic architecture as other measures of intelligence. We used both SNP-based and gene-based GWAS to maximise our ability to discover loci and genes associated with intelligence, before predicting phenotypic intelligence in an independent sample using polygenic profile scoring. We used functional mapping and annotation of genetic associations (FUMA) to identify and annotate independent associations within our data. Finally, we applied gene-set analysis, using 10,891 gene sets sourced from Gene Ontology [27], Reactome [28], and MSigDB [29] to derive biological meaning from our data. Our results indicated that, by drawing on multiple large GWAS datasets all measuring intelligence-related traits, we could attain greater statistical power to detect genetic variants associated with intelligence, facilitate our understanding of the underlying biology of intelligence differences, and make substantial phenotypic predictions of intelligence using SNP data.

## Method

### Samples

Summary statistics were obtained from GWAS meta-analyses of intelligence (*n* = 78,308) [16], and education (*n* = 329,417) [25]. To maximise sample size in our intelligence dataset, four additional GWASs were performed on the verbal-numerical reasoning (VNR) test in UK Biobank. The VNR test consists of 13 items, 6 verbal and 7 numerical questions, all of which are multiple choice. An individual’s verbal numerical reasoning score was measured by summing the number of correct responses given within a 2 minute time period. Participants performed the VNR test either online or at a UK Biobank assessment centre, with some participants taking the VNR test at multiple time points. If participants took the VNR test at multiple time points, only the earliest was used, leading to four GWASs being performed on VNR (time 1, *N* = 76,051, time 2, *N* = 9266, time 3, *N* = 2552, online, *N* = 33,065). UK Biobank participants who were included in the GWAS from Sniekers et al. [16]. were omitted from the current GWASs. Following quality control, a total of 120,934 new UK Biobank participants were available for GWAS. Ethical approval for UK Biobank was received from the Research Ethics Committee (REC reference 11/NW/0382). This work was conducted under UK Biobank application 10279.

In order to derive genetic correlations between intelligence (and the proxy phenotype of education, as well as the final meta-analytic sample) and health-related and other traits, we used summary statistics from 29 GWAS datasets. Supplementary Table 1 shows the datasets used and provides a reference and sample size for each dataset used.

### UK biobank genotyping

Full details of the UK Biobank genotyping procedure are available elsewhere [30]. Briefly, two custom genotyping arrays were used to genotype 49,950 participants (UK BiLEVE Axiom Array) and 438,427 participants (UK Biobank Axiom Array) [30]. Genotype data on 805,426 markers were available for 488,377 of the individuals in UK Biobank. Imputation was carried out using a combination of the Haplotype Reference Consortium (HRC) reference panel, 1000 genomes, and UK10k. Here, we restrict the analysis to the HRC panel, as advised by UK Biobank. This led to 39,131,578 autosomal SNPs being available for the 120,934 participants who had taken the VNR test [30]. Allele frequency checks [31] were performed against the HRC [32] and 1000G [33] site lists, and variants were removed if the allele frequencies differed from the reference set by more than ±0.2.

Additional quality control steps were implemented in the present study and included the removal of participants with non-British ancestry (identified by Bycroft et al. [30]. by performing a principal component analysis on the genotyped SNP data to remove ethnic outliers from a subset of the UK Biobank participants who self-identified as White British) as well as those with extreme scores based on heterozygosity (extreme scores were defined as those with a principal component-adjusted heterozygosity score above 0.19 as shown by Bycroft et al. [30].) and >5% missingness. Individuals whose reported sex was inconsistent with genetically inferred sex were also removed, as well as individuals with neither XX nor XY chromosomes. Finally, those individuals with >10 putative third degree relatives, identified by Bycroft et al. [30] by estimating the kinship coefficients for all pairs of samples using the software KING [34], were removed. This left 408,095 individuals. Using GCTA [9] on 131,790 reportedly-related participants one from each pair of related individuals was removed, based on a genetic relationship threshold of 0.025, leaving 332,050 individuals. Finally, individuals whose genetic and VNR data were available for analysis in the first wave of genetic data release from UK Biobank were removed as these individuals were already a part of the Sniekers et al. [16] dataset. Following these quality control steps, a sample size of 120,934 individuals was available for the VNR test. SNPs with a minor allele frequency (MAF) < 0.0005, and an imputation quality score < 0.1 were removed along with non-bi-allelic SNPs, resulting in 18,485,882 autosomal SNPs.

### Statistical analysis

#### Association analysis

VNR was analysed separately at one of four time points: three of these were at an assessment centre, VNR 1, VNR 2, VNR MRI, and one was online, VNR Online. All VNR scores were adjusted for age, sex, assessment centre, genotype batch, array, and 40 principal components. Association analysis was performed using an additive model implemented using BGENIE [30].

#### Meta-analysis

MTAG results are susceptible to bias and a large false discovery rate when analyzing sets of GWAS summary statistics where some sets are much more highly-powered than others [26]. In order to improve the statistical power to detect association in the Sniekers [16] data, we first meta-analysed the Sniekers dataset with the four GWASs performed on UK Biobank’s VNR test. This lead to the inclusion of 120,934 new participants. The summary statistics from the four UK Biobank VNR GWASs were meta-analysed with the summary statistics available from Sniekers et al. [16] using a sample size weighted meta-analysis conducted with METAL [35]. This resulted in an intelligence dataset containing 199,242 participants.

#### Multi-trait analysis of genome-wide association studies (MTAG)

MTAG [26] allows the meta-analysis of different traits that are genetically correlated with each other in order to increase power to detect loci in any one of the traits. Only summary data are required in order to carry out MTAG and, as bivariate LD score regression is carried out as part of an MTAG analysis to account for (possibly unknown) sample overlap between the GWAS results, these summary statistics need not come from independent samples. Our goal was to increase the power to detect loci associated with intelligence, and so the meta-analytic results of the GWAS on intelligence by Sniekers et al. [16] and the new participants from UK Biobank were used as our primary GWAS data sets. In order to add power to this combined intelligence dataset, the genetically-correlated proxy phenotype of years of education [25] (*n* = 329,417) was included. MTAG was run using the default settings.

#### Identification of independent genomic loci and functional annotation

Using the meta-analytic dataset produced by MTAG, genetic loci related to intelligence were identified using FUMA [36]. First, independent significant SNPs were identified. Independent significant SNPs were selected on the basis of their *P*-value being genome wide significant (*P* < 5 × 10^−8^) and being independent from each other (*r*^2^ < 0.6) within a 1 mb window. Secondly, SNPs that were in LD of the independent lead SNPs (*r*^2^ ≥ 0.6) within a 1 mb window, and within 1000 genomes reference panel with a MAF of greater than 0.01 were included for further annotation. Thirdly, lead SNPs were identified as a subset of the independent significant SNPs (defined as above). Lead SNPs were defined as independent significant SNPs that were in LD with each other at *r*^2^ < 0.1, again with a 1 mb window. Fourthly, genomic risk loci were identified by merging lead SNPs if they were closer than 250 kb apart, meaning that a genomic risk locus could contain multiple independent significant SNPs and multiple lead SNPs. Finally, all SNPs in LD of *r*^2^ ≥ 0.6 with one of the independent significant SNPs formed the border or edge of the genomic risk loci. To map LD, the 1000 genomes phase 3 was used [33].

Functional annotation was carried out in FUMA [36] using all SNPs found within the independent genomic loci which were in LD of *r*^2^ ≥ 0.6, were nominally significant, and had a MAF of 0.01. To gauge the functional consequences of genetic variation at these SNPs they were first matched based on chromosome, base pair position, reference, and non-reference alleles to a database containing functional annotations including the ANNOVAR categories [37], combined annotation dependent depletion (CADD) scores [38], Regulome DB (RDB) scores [39], and chromatin states [40–42].

The ANNOVAR [37] categories were used to identify the function of the SNP, and to locate its position within the genome. CADD scores are a continuous measurement used to determine how deleterious genetic variation at the SNP is to protein structure and function. Higher scores are indicative of a more deleterious variant, with scores of greater than 12.37 providing evidence of pathogenicity [38]. A Regulome DB score is a categorical measurement based on data from expression quantitative trait loci (eQTLs) as well as chromatin marks. The RDB score ranges from 1a to 7 with lower scores given to the variants with the greatest evidence for having regulatory function.

Chromatin states indicate the level of accessibility of genomic regions. This level of accessibility was described using a 15 point scale predicated for each variant using a hidden Markov model based on five chromatin marks for 127 epigenomes in the Roadmap Epigenomics Project [41]. The lower the chromatin score the greater the level of accessibility to the genome at this site, with scores of less than 8 indicative of an open chromatin region. The minimum chromatic state across tissues was used.

#### Gene-based GWAS

Gene-based analysis was conducted using multi-marker analysis of genomic annotation (MAGMA) [43]. SNPs that were located within protein coding genes were used to derive a *P*-value describing the association found with intelligence. Gene locations and boundaries were used from the NCBI build 37 and LD was controlled for using the 1000 genomes phase 3 release [44]. A Bonferroni correction was applied to control for the multiple tests performed on the 18,199 autosomal genes available for analysis.

#### Tissue type gene expression

In order to identify the importance of particular tissue types relevant to individual differences in intelligence, a gene property analysis was conducted using MAGMA. The goal of this analysis was to determine if, in 30 broad tissue types, and 53 specific tissues, tissue-specific differential expression levels were predictive of the association of a gene with intelligence. Tissue types were taken from the GTEx v6 RNA-seq database [45] with expression values being log2 transformed with a pseudocount of 1 after winsorising at 50 with the average expression value being taken from each tissue. Multiple testing was controlled for using Bonferroni correction for 30, and 53 tests.

#### Gene-set analysis

Gene-set analysis was conducted using MAGMA [43] using competitive testing. A total of 10,891 gene-sets (sourced from Gene Ontology [27], Reactome [28], and, MSigDB [29]) were examined for enrichment in intelligence. A Bonferroni correction was applied to control for the multiple tests performed on the 10,891 gene sets available for analysis.

#### Genetic correlations

In order to address whether the genetic architecture of the meta-analysis of correlated traits conducted here produced a phenotype with the same genetic architecture as intelligence, we derived genetic correlations across 29 cognitive, socio-economic status (SES), mental health, metabolic, anthropometric, reproductive, and health and wellbeing phenotypes. We used linkage disequilibrium score regression [12] to test whether each dataset had sufficient evidence of a polygenic signal, indicated by a heritability *Z*-score [12] of >4 and a mean *χ*^2^ statistic of >1.02 [12]. A minor allele frequency cut-off of <0.01 was applied. Only SNPs that were in HapMap 3 with MAF > 0.05 in the 1000 Genomes EUR reference sample were included. Next, Indels and structural variants were removed as were strand ambiguous variants. SNPs whose alleles did not match those in the 1000 Genomes were also removed. The presence of outliers can increase the standard error in LD score regression [12], and so SNPs where *χ*^2^ > 80 were removed. LD scores and weights for use with European populations were downloaded from (http://www.broadinstitute.org/~bulik/eur_ldscores/). False discovery rate (FDR) was controlled for using Benjamini-Hochberg [46] procedure to control for the 30 tests performed (Alzheimer’s disease was included twice) against each phenotype. The corrected alpha level corresponding to an FDR of 5% was 0.0374 for education, 0.0305 for intelligence in the Sniekers et al. [16]. dataset, and 0.0168 for the final meta-analytic dataset on intelligence [46]. In the case of Alzheimer’s disease, a region encompassing 500 kb on each side of *APOE* was removed and the analysis re-run in order to ensure that the large effects in this region did not bias the regression.

#### Partitioned heritability

Partitioned heritability was carried out using stratified linkage disequilibrium score regression [47]. The goal of the partitioned heritability analysis was to determine if SNPs that explain variance in intelligence cluster in functional regions of the genome. A full description of how this method works can be found in Finucane et al. [47]. Firstly, heritability for each of the functional groupings is derived. Secondly, this heritability estimate is used to derive an enrichment metric defined as the proportion (Pr) of heritability captured by the functional annotation, over the proportion of SNPs contained within it (Pr(h^2^)/Pr(SNPs)). This ratio describes whether a functional annotation contains a greater or lesser proportion of the heritability than would be predicted by the proportion of SNPs it contains, Pr(h^2^)/Pr(SNPs) = 1. The proportion of the heritability of each category is used as the numerator, rather than the heritability of each category. Stratified LD Scores were calculated from the European-ancestry samples in the 1000 Genomes project (1000G) and only included the HapMap 3 SNPs with a minor allele frequency (MAF) of >0.05. A model was derived using 52 overlapping, functional categories. Correction for multiple testing was performed using a Bonferroni test on the 52 functional categories (*α* = 0.00096).

#### Genetic prediction

The three smaller samples of individuals who performed the VNR test and had their genetic data released in the second release of the UK Biobank genetic data were used. The METAL meta-analysis and MTAG meta-analysis with education were re-run leaving out one of the groups who performed the VNR test. The group that was left out were then used as the target sample for polygenic prediction. Using our meta-analytic dataset on intelligence, polygenic risk scores were derived for intelligence in each of the VNR groups using PRSice [48]. SNPs that were strand ambiguous and those with a MAF of <0.01 were removed prior to deriving the polygenic risk scores. SNPs were clumped using the binary.ped files from the participants in the UK Biobank as a reference (*r*^2^ < 0.25, 250 kb window). Polygenic scores were then derived for each participant as the sum of alleles associated with intelligence, weighted by the effect size from our meta-analytic intelligence dataset. A total of five polygenic risk scores were derived using the following *P*-value cut offs: 0.01, 0.05, 0.1, 0.5, and 1.

## Results

Genetic correlations performed for each of the four VNR groups whose genetic data were released in the second release of the UK Biobank genetic data against the Sniekers dataset [16] indicated no evidence of sample overlap; this is shown by there being an intercept of around zero for each comparison. By meta-analysing the four VNR groups with the Sniekers data we were able to increase the mean *χ*^2^ of the Sniekers dataset from to 1.30 to 1.59, making it similar in statistical power to the Okbay education dataset [25] (mean *χ*^2^ = 1.65).

**Fig. 1:**
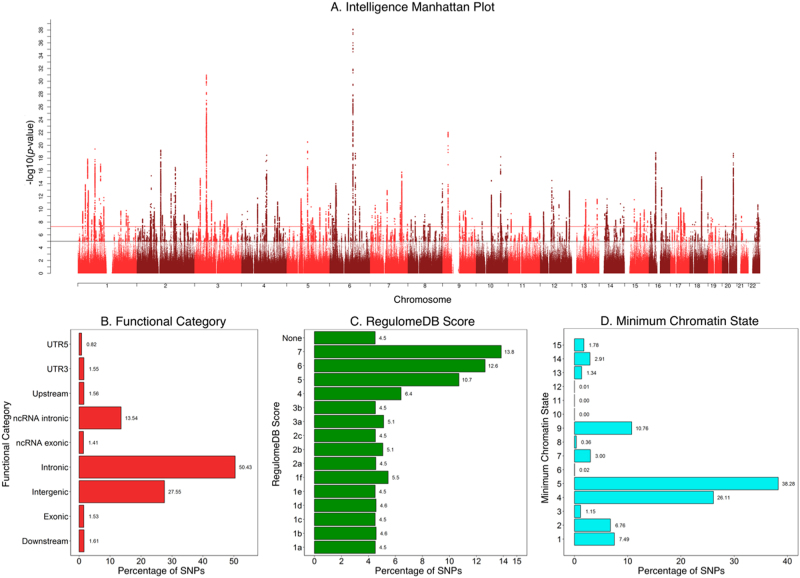
**a**. The results of our MTAG analysis. SNP-based GWAS Manhattan plot; negative log10 transformed *P*-values for each SNP are plotted against chromosomal location. The red line indicates genome-wide significance and the black line indicates suggestive associations. **b**. Functional annotation carried out using FUMA on the independent genomic loci identified. The percentage of SNPs found in each of the nine functional categories is listed. **c**. The percentage of SNPs from the independent genomic loci that fell into each of the Regulome DB scores categories. A lower score indicates greater evidence for that SNPs involvement in gene regulation. **d**. The percentage of SNPs within the independent genomic loci plotted against the minimum chromatic state for 127 tissue/cell types

Using MTAG to combine our GWAS of intelligence with those of education [25], we were able to increase the mean *χ*^2^ in the intelligence dataset from 1.59 to 1.73. This corresponds to an increase in the sample size from 199,242 to 248,482. The maxFDR was calculated using the same procedure as Turley et al. [26] whereby assuming that at least 10% of SNPs were causal for each trait, an FDR of 0.0004 was derived for intelligence. The MTAG analysis that combined these two GWASs found 11,930 genome wide significant SNPs associated with intelligence (Fig. 1a). These SNPs were found in 187 independent loci, identified using FUMA (Supplementary Table 2); [49, 36] this represents an increase of 169 loci compared to those reported in the Sniekers et al. GWAS alone [16]. In order to determine if differences in loci construction were influencing the difference across the intelligence (Sniekers), education (Okbay), and present study, we performed FUMA (using the same parameters as in the current study) on the Sniekers and Okbay datasets and compared the loci found across phenotypes. Within the publically available Okbay dataset, 77 genome wide significant loci were identified and, 25 of these 77 loci were not associated in our meta-analytic dataset (Supplementary Table 3) and are unique to education, providing evidence that MTAG does not simply find the genetic average between two traits. Upon examination of the Sniekers dataset, only 16 loci were found rather than the 18 reported, a difference most likely caused by the use of the unusually small window (300 kb) used for clumping by Sniekers.

Comparing the genomic loci identified using FUMA in the current study to the 16 loci identified from Sniekers et al. [16] using FUMA, only one locus on chromosome 15 was found in Sniekers that was not present in the current study. A total of 130 of the 187 loci reported in the current study are novel and not reported previously with intelligence or education (Supplementary Table 3). By comparing the genomic loci found in the present study with the same SNPs in the intelligence and education data sets used in its construction, we see low *P*-values across each of the three, consistent with the finding of a strong genetic correlation between each of the three phenotypes (Supplementary Table 4).

Using LD Score regression [50], a heritability estimate of 25.44% (SE = 0.84%) was found for our MTAG analysis of intelligence. There was no evidence of residual stratification or confounding leading to an inflation of test statistics (LD Score regression intercept = 0.98).

Functional annotation conducted in FUMA indicated that, across the independent genomic loci associated with intelligence, there was an overrepresentation of SNPs found in introns (50%), as well as SNPs found in intergenic regions (28%) (Fig. 1b, Supplementary Table 2). There was also evidence that these loci contained regulatory regions of the genome, indicated by 28% of the SNPs in the genomic loci having Regulome DB scores with less than 2 providing evidence that genetic variation at this SNP is likely to affect gene expression (Fig. 1c, Supplementary Table 2). Finally, a total of 83% of the SNPs within the genomic loci had a minimum chromatin state of <8 indicating that they are located in an open chromatin state, providing additional evidence that they are located within regulatory regions (Fig. 1d, Supplementary Table 2). Finally, 65% of the SNPs within the genomic loci showed evidence of being an eQTL, and 3.79% had a CADD score of greater than 12.37 indicating that variation at these SNPs is deleterious (Supplementary Table 2).

**Fig. 2:**
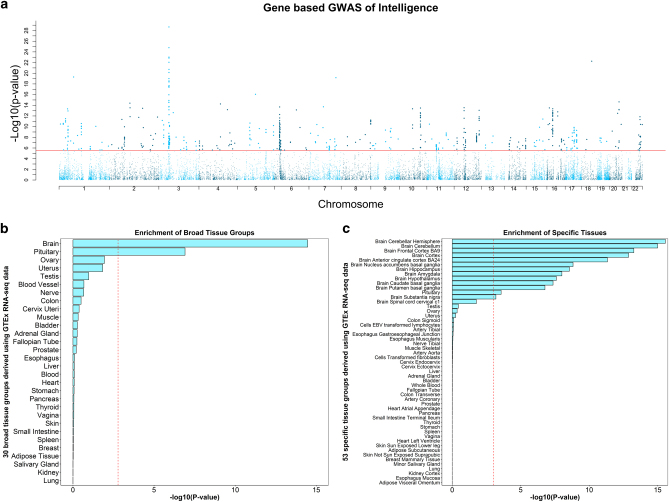
**a**. Gene based Manhattan plot; negative log10 transformed *P*-values for each gene (derived using MAGMA) are plotted against chromosomal location. The red line indicates genome wide significance. **b** gene property analysis linking transcription differences in 30 broad tissue types (*y*-axis) with the gene based statistics produced from MAGMA. Red line indicates significance following Bonferroni correction for the 53 tests performed. **c** gene property analysis linking transcription differences in 53 tissue types (*y*-axis) with the gene based statistics produced from MAGMA. Red line indicates significance following Bonferroni correction for the 53 tests performed

A gene-based GWAS was conducted using MAGMA. Gene based analysis can increase power to detect significant associations as the signal across many SNPs (all within a gene) is combined [51]. A total of 538 (Fig. 2a, Supplementary Table 5) genes attained genome-wide significance using a gene-based GWAS.

The results of the gene property analysis, conducted using MAGMA and linking transcription differences in 30 broad tissues with intelligence differences, found a significant relationship between intelligence and expression changes in the brain (*P* = 3.39 × 10^−15^), and the pituitary (*P* = 1.23 × 10^−7^) (Fig. 2b, Supplementary Table 6). An examination of 53 tissue specific gene sets showed that this relationship between transcription changes in the brain and intelligence was evident across cortical tissue types including cerebellar hemisphere (*P* = 2.76 × 10^−16^), the cerebellum (*P* = 1.00 × 10^−15^), the frontal cortex (*P* = 5.47 × 10^−14^), the anterior cingulate cortex (*P* = 4.61 × 10^−12^), the nucleus accumbens of the basal ganglia (*P* = 1.50 × 10^−9^), hippocampus (*P* = 2.77 × 10^−9^), amygdala (*P* = 9.80 × 10^−9^), hypothalamus (*P* = 2.51 × 10^−8^), caudate nucleus (*P* = 4.35 × 10^−8^), putamen (*P* = 1.69 × 10^−7^), and the substantia nigra (*P* = 6.46 × 10^−4^) (Fig. 2c, Supplementary Table 7). These results are the first to report that transcription differences in cortical tissues are linked with individual differences in intelligence.

In order to obtain information on the biological systems involved in intelligence differences that are influenced by genetic variation, we conducted gene-set analysis using all genes available irrespective of their level of association. Using a competitive test of enrichment implemented in MAGMA, we identified seven novel biological systems associated with intelligence differences (Table 1, Supplementary Table 8). Firstly, we identify a role for neurogenesis (gene-set size = 1,355 genes, *P*-value = 5.59 × 10^−10^), the process by which neurons are generated from neural stem cells. Secondly, a role was also found for genes expressed in the synapse (gene-set size = 717 genes, *P*-value = 1.43 × 10^−6^), consistent with previous studies showing a role for synaptic plasticity [52]. Thirdly, enrichment was found for the regulation of nervous system development (gene-set size = 722 genes, *P*-value = 4.02 × 10^−8^). Fourthly, we find evidence for enrichment for neuron projection (gene-set size = 898 genes, *P*-value = 2.07 × 10^−7^), neuron differentiation (gene-set size = 842 genes, *P*-value = 1.62 × 10^−6^), and central nervous system neuron differentiation (gene-set size = 160 genes, *P*-value = 5.33 × 10^−7^). Finally, we identify a role for oligodendrocyte differentiation (gene-set size = 1037 genes, *P*-value = 1.75 × 10^−6^). In addition to these novel results, the finding that regulation of cell development (gene-set size = 808 genes, *P*-value 9.71 × 10^−7^) is enriched for intelligence was replicated [16].

**Table 1:** Gene-sets attaining statistical significance following Bonferroni control for multiple tests

Gene-set Name	Number of genes in gene set	Beta	SE of Beta	P-value
Neurogenesis	1355	0.20	0.05	5.59 × 10^−10^
Regulation of nervous system development	722	0.23	0.05	4.02 × 10^−8^
Regulation of cell development	808	0.22	0.04	7.38 × 10^−8^
Neuron projection	898	0.20	0.04	2.07 × 10^−7^
Central nervous system neuron differentiation	160	0.47	0.04	5.33 × 10^−7^
Synapse	717	0.21	0.04	1.43 × 10^−6^
Neuron differentiation	842	0.19	0.04	1.62 × 10^−6^
Oligodendrocyte differentiation	1037	0.17	0.04	1.75 × 10^−6^

**Fig. 3:**
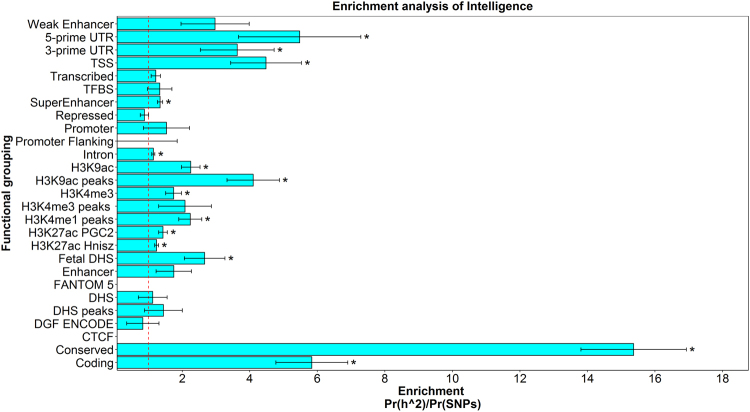
Enrichment analysis for intelligence using the 52 functional categories. This analysis differs from that performed by FUMA as all SNPs are used whereas, in FUMA, only those in the independent genomic loci are annotated. The enrichment statistic is the proportion of heritability found in each functional group divided by the proportion of SNPS in each group (Pr(h^2^)/Pr(SNPs)). The dashed line indicates no enrichment found when Pr(h^2^)/Pr(SNPs) = 1. Statistical significance is indicated by asterisk

Partitioned heritability analysis indicated the functional regions of the genome that make a greater contribution to intelligence differences than would be expected based on the proportion of SNPs captured by the groupings. We find, for the first time, that coding regions were significantly enriched for the heritability of intelligence (*P* = 1.59 × 10^−5^), as were transcriptional start sites (*P* = 9.19 × 10^−4^). We also find enrichment of heritability for the histone marks of H3K9ac (*P* = 7.81 × 10^−6^), H3K4me1 (*P* = 1.43 × 10^−5^), H3K27ac PGC2 (*P* = 9.16 × 10^−4^), and H3K27ac Hnisz (*P* = 3.09 × 10^−5^). We also replicate the finding that regions of the genome that have undergone purifying selection are enriched for intelligence [23] (*P* = 7.77 × 10^−16^) (Fig. 3, Supplementary Table 9).

Using our meta-analytic dataset on intelligence we carried out polygenic prediction into UK Biobank subsamples following their removal from the meta-analysis. Between 3.64 and 6.84% of phenotypic intelligence (as measured by the VNR Test in UK Biobank) could be predicted (Supplementary Table 10); the upper limit is an improvement of ~43% on the largest reported estimate to date, of 4.8% [16]. The polygenic risk scores that predicted the greatest amount of variance were those composed of the *P* < 0.05, and *P* < 0.1 cut off in the VNR MRI group. However, a highly similar *r*^2^ was also evident at higher *P*-value thresholds indicating that, despite our increase in power, many of the genetic variants associated with intelligence can still be found across the full distribution of *P*-values.

**Fig. 4:**
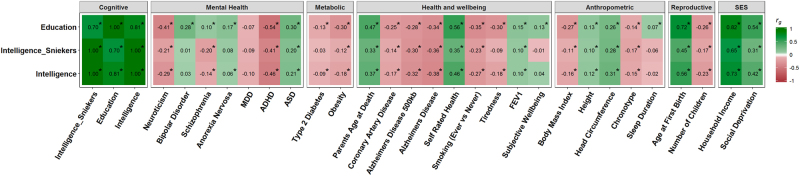
Heat map showing the genetic correlations between the meta-analytic intelligence phenotype, intelligence, education with 29 cognitive, SES, mental health, metabolic, health and wellbeing, anthropometric, and reproductive traits. Positive genetic correlations are shown in green and negative genetic correlations are shown in red. Statistical significance following FDR (using Benjamini-Hochberg procedure [51]) correction is indicated by an asterisk

We next derived genetic correlations with 29 phenotypes both to obtain evidence suggesting that the results of our meta-analysis produced a phenotype with the same genetic architecture as intelligence, and to examine additional phenotypes that might be genetically correlated with intelligence (Fig. 4, Supplementary Table 11). Many of these have been shown before using intelligence phenotypes [11, 12], and replicated using the verbal-numerical reasoning phenotype from UK Biobank; [53] however, we include them to show the similarities and differences between the genetic architecture found in our meta-analytic intelligence dataset and the three datasets used in its construction. A heritability *Z*-score of 30.29 was found for our meta-analytic intelligence dataset and a mean *χ*^2^ of 1.73 indicating a sufficient level of polygenicity within the dataset for use with LD regression [12]. We find a novel genetic correlation between intelligence and parental longevity; this is found using the intelligence [16] GWAS (*r*_*g*_ = 0.33, SE = 0.08) and our meta-analytic sample (*r*_*g*_ = 0.37, SE = 0.07). This indicates that the polygenic load for greater intelligence is associated with greater longevity, using parental longevity as a proxy phenotype.

When compared with previous GWASs, our meta-analytic dataset showed strong positive genetic correlations with each of the two variables measured: intelligence (Sniekers, *r*_*g*_ = 1.00, SE = 0.01); and years of education (*r*_*g*_ = 0.81, SE = 0.009). The genetic correlation of 1 between our intelligence dataset and that of Sniekers further indicates that the underlying polygenic signal in our meta-analytic dataset is highly similar to that of intelligence, rather than being an average between education and intelligence (Fig. 4). For the SES variables the point estimate of the genetic correlation with our meta-analytic intelligence dataset fell between that of the intelligence [16] GWAS and the education GWAS [25] which is a trend seen across some of the traits assessed.

For the mental health variables, our meta-analytic intelligence dataset showed a pattern of genetic correlations more similar to Sniekers [16] GWAS on intelligence than the Okbay [25] GWAS on education. For bipolar disorder, no genetic correlation was found using our meta-analytic dataset or with the Sniekers dataset; however, a genetic correlation was found with education (*r*_*g*_ = 0.28, SE = 0.04). For bipolar disorder, previous results have indicated a negative genetic correlation using established measures of intelligence, although after correcting for multiple tests this estimate was not statistically significant [11]. Similar results were also found when examining schizophrenia, where a positive genetic correlation was found with education (*r*_*g*_ = 0.10, SE = 0.02), and a negative genetic correlation was found with both intelligence datasets (Sniekers, *r*_*g*_ = −0.20, SE = 0.03, current study *r*_*g*_ = −0.14, SE = 0.02).

Differences between the previous GWAS on intelligence [16] and our meta-analysis were also evident for tiredness, anorexia nervosa, and type 2 diabetes. For these phenotypes, the point estimate of the genetic correlation is indistinguishable from zero for the intelligence [16] GWAS but significant and in the same direction for both education and intelligence in our meta-analytic sample.

## Discussion

People with a higher level of cognitive function have been observed to have better physical and mental health, and to have longer lives [3, 7]. This paper exploited the high genetic correlations found between intelligence and education, increased the statistical power of a GWAS on intelligence, and attempted to find the loci and biological mechanisms that help explain intelligence differences, and the health differences with which they are associated. Through the use of summary statistics drawn from a large GWAS on intelligence and education, and the latest release of the UK Biobank genetic data used in conjunction with a recently-developed method, MTAG [26], we were able to assemble the sample sizes required to achieve the high levels of statistical power needed to detect loci of small effect that explain differences in intelligence. These analyses produced a number of novel findings.

First, we found 187 independent associations for intelligence in our GWAS, and highlighted the role of 538 genes being involved in intelligence, a substantial advance on the 18 loci previously reported [16]. Within the 187 loci, we found clear evidence of functionality, indicated by our ability to link these SNPs to open chromatin states and regulatory elements of the genome, and by the finding that many of the loci contained regions where genetic variation was deleterious.

Second, using two strategies, we uncovered additional functional elements of the genome associated with intelligence differences. Both of these methods used the whole polygenic signal in our final dataset rather than only the most significant regions as used in FUMA. Using MAGMA, we found that transcription differences in the brain and pituitary gland were associated with intelligence. This relationship with cortical tissues was found across the cortex and in multiple cortical tissues (Fig. 2b and c). Using stratified linkage disequilibrium score regression, we replicated the finding that regions of the genome that have undergone purifying selection were the most strongly associated with intelligence differences [23]. We also found that coding regions and histone marks are enriched for intelligence-associated regions of the genome.

Third, we used our meta-analytic GWAS data to predict almost 7% of the variation in intelligence in one of three independent samples. The range of similar estimates across the three independent samples was 3.6 to 6.8%. Previous estimates of prediction have been ∼5% at most; [16] our results thus indicate that prediction accuracy can be improved by drawing on existing data sets of proxy phenotypes for intelligence, as we did here. Additionally, polygenic profile scores derived using the MTAG method can be used to make meaningful predictions regarding an individual level of intelligence.

Fourth, we report the novel finding that the polygenic signal across our GWAS dataset clusters in genes involved in the process of neurogenesis, genes expressed in the synapse, and genes involved in the development of the nervous system, as well as those involved in myelination within the central nervous system due to their role in oligodendrocyte differentiation. This provides a rationale for a theory of how genetic differences, via their influence on physiological differences, contribute to variation in intelligence.

The finding of neurogenesis gene-set enrichment for intelligence is persuasive, because neurogenesis has been linked to cognitive processes—particularly pattern separation and cognitive flexibility—in rodent models. New neurons are continually made in humans in the subgranular zone of the hippocampus and in the striatum; [54] in rodent studies, experimentally reducing analogous neurogenesis results in a poorer ability to discriminate between highly similar patterns [55], whereas increasing the number of new neurons produced results in an increased ability to successfully discriminate between highly similar stimuli [56]. Additionally, neurogenesis appears to be involved in cognitive flexibility by serving to avoid interference between novel and previously formed memories in a spatial navigation task [57, 58]. Such findings have been expanded to include touch-screen discrimination tasks [59], as well as active place avoidance [60]. Across these experiments, the common finding was that neurogenesis was not required for the learning of the task, but rather for the reversal of the rule once the formally correct response had changed, suggesting that neurogenesis is an important mechanism in cognitive flexibility. Replication of this finding of enrichment of neurogenesis in intelligence GWAS data—in an independent sample—is required to confirm our finding of a biological mechanism associated with intelligence differences in humans.

Oligodendrocyte differentiation was also identified by gene-set analysis as being involved in intelligence differences. The central nervous system of humans contains a very high percentage (~50%) of white matter, which is maintained by the action of oligodendrocytes [61]. Abnormalities in white matter are also associated with psychiatric disorders such as schizophrenia and autism [60], conditions that have previously been shown to be genetically linked to differences in intelligence. By finding that genes involved in the myelination of the central nervous system are associated with cognitive variation, we provide a molecular genetic basis for the link between white matter tract structure and intelligence [62].

Finally, we showed, using genetic correlations with 29 other traits, that our meta-analytic intelligence GWAS had a highly similar genetic architecture to that of intelligence alone. The genetic correlations that were produced using the meta-analytic intelligence GWAS did differ for some traits; this was most evident for schizophrenia, for which positive genetic correlations have been observed with education [12], but negative associations with intelligence [11]. Our new findings provide evidence that the previously-discovered differences in genetic correlations between traits such as schizophrenia and intelligence and education [11, 57, 12] are due to the fact that genetic effects acting solely on intelligence are those that are negatively genetically correlated with schizophrenia, indicating they are protective against the disorder. However, the genetic variants that act on both education and intelligence are those that show positive genetic correlations with schizophrenia. By meta-analysing intelligence with the genetic component of education that overlaps with intelligence, the relative contribution of variance that is unique to intelligence lessens, and so too does the magnitude of the genetic correlation with schizophrenia.

This limitation—a greater proportion of variance that is common across education and intelligence in our results—has implications for the results of our GWAS, since those variants that gain the most signal from meta-analysis across genetically correlated traits will, by definition, show association with each trait in our meta-analysis. Whereas the final results of our GWAS did indicate the loci that are likely to be involved in intelligence differences, our GWAS may be overrepresented by effects that are also associated with education. Nevertheless, we did find associations on each chromosome that were not found in the most recent GWAS of education [25], and peaks that were identified as being associated with education on chromosomes 1, 2, 3, 4, 5, 6, 7, 9, 11, 12, 13, 14, 15, 16, and 18 [25] were not found to be genome-wide significant in our meta-analytic dataset (Supplementary Table 2). These differences cannot be explained by using different criteria for loci discovery, as we used the same software (FUMA) with the same parameters. It should also be noted that, whereas the genetic correlation of our meta-analytic dataset with education did increase from *r*_*g*_ = 0.70 to *r*_*g*_ = 0.80, the genetic correlation with intelligence from the Sniekers dataset remained at *r*_*g*_ = 1.00, showing that the MTAG procedure produces a phenotype with a highly similar genetic architecture to the trait of interest.

Future work using large GWAS that are exclusively based on established tests of intelligence [21] will provide valuable samples in which to attempt replication of these findings. The strength of the MTAG approach used here, drawing power from related phenotypes, lies in the accumulation of additional power to detect loci, make more accurate predictions based on SNP data, and the ability to identify the biological significance behind the polygenic signal in such data sets.

## Electronic supplementary material

Supplementary Table 1

Supplementary Tables 2-7
