# Supplementary material for: A combined analysis of genetically correlated traits identifies 187 loci and a role for neurogenesis and myelination in intelligence
Source: Mol Psychiatry. 2018 Jan 11;24(2):169–81. doi: 10.1038/s41380-017-0001-5 (PMC6344370; doi:10.1038/s41380-017-0001-5)
Supplement: Supplementary file 1 — Supplementary Table 1 [file 41380_2017_1_MOESM1_ESM.docx]

**Supplemental Table 1**. The source of the GWAS data sets used for genetic correlations

|  | **Phenotype** | **Consortium** | **Reference** | **No. of individuals in GWAS** |
| --- | --- | --- | --- | --- |
| Used in MTAG | Intelligence^1^ | - | Sniekers et al. Nature Genetics (2017); advanced online publication. PMID:28530673 | 78 308 |
|  | Education^2^ | SSGAC | Okbay et al. Nature (2016);7604: 539-542. PMID: 27225129 | 329 417 |
|  |  |  |  |  |
| Used to derive genetic correlations |  |  |  |  |
|  |  |  |  |  |
|  | Household Income^3^ | - | Hill, et al. Current Biology (2016); 22:3083-3089. PMID: 27818178 | 96 900 |
|  | Social Deprivation^3^ |  | Hill, et al. Current Biology (2016); 22:3083-3089. PMID: 27818178 | 112 005 |
|  | Parents’ age at Death^4^ | - | Pilling et al. Aging (2016); 8:1-24. PMID: 27015805. | 75 244 |
|  | ADHD | Psychiatric Genetics Consortium (PGC) | Demontis et al. (2017); BioRxiv. | 19,099 cases 34,194 controls |
|  | Bipolar disorder^5^ | Psychiatric Genetics Consortium (PGC) | Psychiatric GWAS Consortium Bipolar Disorder Working Group. Nature Genetics (2011); 43: 977-983. PMID: 21926972 | 7481 cases  9250 controls |
|  | Schizophrenia^6^ | Psychiatric Genetics Consortium (PGC) | Schizophrenia Working Group of the Psychiatric Genomics Consortium. Nature (2014); 511: 421-427. PMID: 25056061 | 36 989 cases  113 075 controls |
|  | MDD^7^ | Psychiatric Genetics Consortium (PGC) | Major Depressive Disorder Working Group of the Psychiatric GWAS Consortium. Molecular Psychiatry (2013); 18: 497-511. PMID: 22472876 | 9240 cases  9519 controls |
|  | ASD^8^ | PGC | Cross-Disorder Group of the Psychiatric Genomics Consortium, Lancet (2013) | 10 226 |
|  | Coronary Artery Disease^9^ | CARDIoGRAM | Schunkert et al. Nature Genetics 2011; 43: 333-338. PMID: 21378990 | 22 233 cases  64 762 controls |
|  | Type 2 diabetes^10^ | DIAGRAM | Morris et al. Nature Genetics (2012); 44: 981-990. PMID: 22885922 | 12 171 cases  56 862 controls |
|  | Obesity^11^ | GIANT | Berndt et al. Nature Genetics 2013; 45:501-512, PMID: | 263 407 |
|  | Alzheimer's disease^12^ | International Genomics of Alzheimer’s Project (IGAP) | Lambert et al. Nature Genetics (2013); 45: 1452-1458. PMID: 24162737 | 17 008 cases  37 154 controls |
|  | Anorexia Nervosa | Genetic Consortium for Anorexia Nervosa (GCAN) | Boraska, Vesna, et al. Molecular Psychiatry (2014); 19(10): 1085-1094. PIMD: 24514567 | 2907 cases 14860 controls |
|  | Self-Rated Health^13^ | - | Harris et al. International Journal of Epidemiology (2016); PMID:27864402 | 111 749 |
|  | Smoking (Ever Vs Never)^14^ | Tobacco and Genetics Consortium | Tobacco and Genetics Consortium. Nature Genetics (2010); 5: 441-447. PMID: 20418890. | 74 053 |
|  | Neuroticism | - | Smith et al. Molecular Psychiatry (2016). 21(6): 749-757 | 106 000 |
|  | Tiredness^15^ | - | Deary et al. Molecular Psychiatry (2017); PMID:28322280. | 108 976 |
|  | Subjective Wellbeing^16^ | - | Okbay et al. Nature Genetics (2016);6:624-633. PMID: 27089181 | 193 397 |
|  | BMI^17^ | GIANT | Locke et al. Nature (2015); 518: 197-206. PMID: 25673413 | 339 224 |
|  | Height^18^ | GIANT | Wood et al. Nature Genetics (2014); 11: 1173-1186. PMID:25282103 | 253 288 |
|  | Head Circumference^19^ | EGG | Taal et al. Nature Genetics (2012);4:532-538. PMID:22504419 | 10 678 |
|  | Chronotype^20^ | - | Jones et al. PLoS Genetics (2016):PMID:27494321 | 128 266 |
|  | Sleep Duration^20^ | - | Jones et al. PLoS Genetics (2016):PMID:27494321 | 128 266 |
|  | Age at First Birth^21^ | SSGAC | Barban et al. Nature Genetics (2016):12;1462-1472.PMID:27798627 | 222 037 |
|  | Number of Children^21^ | SSGAC | Barban et al. Nature Genetics (2016):12;1462-1472.PMID:27798627 | 318 863 |

References

1. Sniekers S, Stringer S, Watanabe K, Jansen PR, Coleman JR, Krapohl E *et al.* Genome-wide association meta-analysis of 78,308 individuals identifies new loci and genes influencing human intelligence. *Nat Genet* 2017.

2. Okbay A, Beauchamp JP, Fontana MA, Lee JJ, Pers TH, Rietveld CA *et al.* Genome-wide association study identifies 74 loci associated with educational attainment. *Nature* 2016; **533**(7604)**:** 539-542.

3. Hill WD, Hagenaars SP, Marioni RE, Harris SE, Liewald DC, Davies G *et al.* Molecular genetic contributions to social deprivation and household income in UK Biobank. *Curr Biol* 2016; **26**(22)**:** 3083-3089.

4. Pilling LC, Atkins JL, Bowman K, Jones SE, Tyrrell J, Beaumont RN *et al.* Human longevity is influenced by many genetic variants: evidence from 75,000 UK Biobank participants. *Aging (Albany NY)* 2016; **8**(3)**:** 547.

5. Psychiatric GWAS Consortium Bipolar Disorder Working Group. Large-scale genome-wide association analysis of bipolar disorder identifies a new susceptibility locus near ODZ4. *Nat Genet* 2011; **43**(10)**:** 977-983.

6. Schizophrenia Working Group of the Psychiatric Genomics Consortium. Biological insights from 108 schizophrenia-associated genetic loci. *Nature* 2014; **511**(7510)**:** 421-427.

7. Ripke S, Wray NR, Lewis CM, Hamilton SP, Weissman MM, Breen G *et al.* A mega-analysis of genome-wide association studies for major depressive disorder. *Mol Psychiatry* 2013; **18**(4)**:** 497-511.

8. Cross-Disorder Group of the Psychiatric Genomics Consortium. Identification of risk loci with shared effects on five major psychiatric disorders: a genome-wide analysis. *Lancet* 2013; **381**(9875)**:** 1371–1379.

9. Schunkert H, König IR, Kathiresan S, Reilly MP, Assimes TL, Holm H *et al.* Large-scale association analysis identifies 13 new susceptibility loci for coronary artery disease. *Nat Genet* 2011; **43**(4)**:** 333-338.

10. Morris AP, Voight BF, Teslovich TM, Ferreira T, Segre AV, Steinthorsdottir V *et al.* Large-scale association analysis provides insights into the genetic architecture and pathophysiology of type 2 diabetes. *Nat Genet* 2012; **44**(9)**:** 981.

11. Berndt SI, Gustafsson S, Mägi R, Ganna A, Wheeler E, Feitosa MF *et al.* Genome-wide meta-analysis identifies 11 new loci for anthropometric traits and provides insights into genetic architecture. *Nat Genet* 2013; **45**(5)**:** 501-512.

12. Lambert J-C, Ibrahim-Verbaas CA, Harold D, Naj AC, Sims R, Bellenguez C *et al.* Meta-analysis of 74,046 individuals identifies 11 new susceptibility loci for Alzheimer's disease. *Nat Genet* 2013; **45**(12)**:** 1452-1458.

13. Harris SE, Hagenaars SP, Davies G, Hill WD, Liewald DC, Ritchie SJ *et al.* Molecular genetic contributions to self-rated health. *bioRxiv* 2015**:** 029504.

14. Tobacco and Genetics Consortium. Genome-wide meta-analyses identify multiple loci associated with smoking behavior. *Nat Genet* 2010; **42**(5)**:** 441-447.

15. Deary V, Hagenaars SP, Harris SE, Hill WD, Davies G, Liewald DC *et al.* Genetic contributions to self-reported tiredness. *bioRxiv* 2016**:** 047290.

16. Okbay A, Baselmans BM, De Neve J-E, Turley P, Nivard MG, Fontana MA *et al.* Genetic variants associated with subjective well-being, depressive symptoms, and neuroticism identified through genome-wide analyses. *Nat Genet* 2016.

17. Locke AE, Kahali B, Berndt SI, Justice AE, Pers TH, Day FR *et al.* Genetic studies of body mass index yield new insights for obesity biology. *Nature* 2015; **518**(7538)**:** 197-206.

18. Wood AR, Esko T, Yang J, Vedantam S, Pers TH, Gustafsson S *et al.* Defining the role of common variation in the genomic and biological architecture of adult human height. *Nat Genet* 2014; **46**(11)**:** 1173-1186.

19. Taal HR, St Pourcain B, Thiering E, Das S, Mook-Kanamori DO, Warrington NM *et al.* Common variants at 12q15 and 12q24 are associated with infant head circumference. *Nat Genet* 2012; **44**(5)**:** 532-538.

20. Jones SE, Tyrrell J, Wood AR, Beaumont RN, Ruth KS, Tuke MA *et al.* Genome-wide association analyses in 128,266 individuals identifies new morningness and sleep duration loci. *PLoS Genet* 2016; **12**(8)**:** e1006125.

21. Barban N, Jansen R, de Vlaming R, Vaez A, Mandemakers JJ, Tropf FC *et al.* Genome-wide analysis identifies 12 loci influencing human reproductive behavior. *Nat Genet* 2016; **48**(12)**:** 1462-1472.
